# Lipid remodeling in acrosome exocytosis: unraveling key players in the human sperm

**DOI:** 10.3389/fcell.2024.1457638

**Published:** 2024-09-23

**Authors:** Laila Suhaiman, Silvia A. Belmonte

**Affiliations:** ^1^ Instituto de Medicina y Biología Experimental de Cuyo (IMBECU)-CONICET (Consejo Nacional de Investigaciones Científicas y Técnicas), Argentina; ^2^ Facultad de Ciencias Médicas, Universidad Nacional de Cuyo, Mendoza, Argentina; ^3^ Instituto de Histología y Embriología de Mendoza (IHEM) “Dr. Mario H. Burgos”, CONICET (Consejo Nacional de Investigaciones Científicas y Técnicas), Universidad Nacional de Cuyo, Mendoza, Argentina

**Keywords:** acrosome exocytosis, human sperm, lipids, cholesterol, sphingolipids, phospholipids, membrane fusion

## Abstract

It has long been thought that exocytosis was driven exclusively by well-studied fusion proteins. Some decades ago, the role of lipids became evident and escalated interest in the field. Our laboratory chose a particular cell to face this issue: the human sperm. What makes this cell special? Sperm, as terminal cells, are characterized by their scarcity of organelles and the complete absence of transcriptional and translational activities. They are specialized for a singular membrane fusion occurrence: the exocytosis of the acrosome. This unique trait makes them invaluable for the study of exocytosis in isolation. We will discuss the lipids’ role in human sperm acrosome exocytosis from various perspectives, with a primary emphasis on our contributions to the field. Sperm cells have a unique lipid composition, very rare and not observed in many cell types, comprising a high content of plasmalogens, long-chain, and very-long-chain polyunsaturated fatty acids that are particular constituents of some sphingolipids. This review endeavors to unravel the impact of membrane lipid composition on the proper functioning of the exocytic pathway in human sperm and how this lipid dynamic influences its fertilizing capability. Evidence from our and other laboratories allowed unveiling the role and importance of multiple lipids that drive exocytosis. This review highlights the role of cholesterol, diacylglycerol, and particular phospholipids like phosphatidic acid, phosphatidylinositol 4,5-bisphosphate, and sphingolipids in driving sperm acrosome exocytosis. Furthermore, we provide a comprehensive overview of the factors and enzymes that regulate lipid turnover during the exocytic course. A more thorough grasp of the role played by lipids transferred from sperm can provide insights into certain causes of male infertility. It may lead to enhancements in diagnosing infertility and techniques like assisted reproductive technology (ART).

## 1 Introduction

During exocytosis, vesicles within the cell move toward and fuse with the plasma membrane. In constitutive secretion, the vesicles provide proteins and lipids that will become components of the cell membrane. Otherwise, regulated exocytosis is accomplished by an intracellular calcium increase, and vesicles usually contain soluble bioactive molecules to be secreted to the extracellular environment. Neurons and endocrine and neuroendocrine cells are highly specialized in releasing active biological compounds. However, regulated exocytosis is a central issue for fertilization. The oocyte and spermatozoon require exocytosis for successful fertilization to proceed. Fertilization implies a meticulously coordinated series of events, leading to the formation of a zygote (Bhakta et al., 2019; Evans and Florman, 2002). Penetration of the oocyte coat, fusion with its plasma membrane, and ultimately fertilization necessitate the secretion of the special sperm vesicle, named acrosome, resulting in the exposure of sperm membranes required to complete the process (Belmonte et al., 2016; Buffone et al., 2014; Tomes, 2007).

**FIGURE 1:**
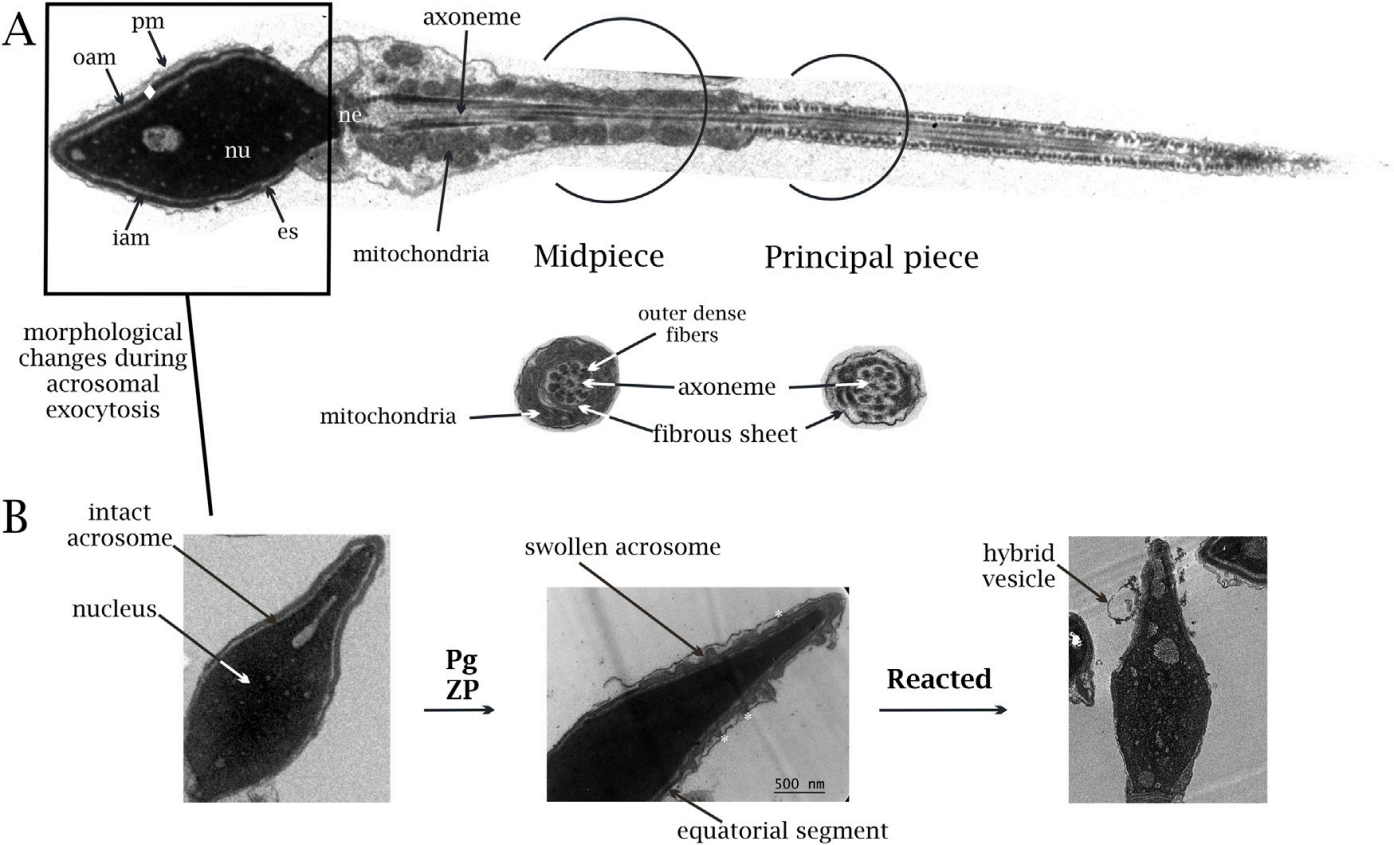
**(A)** Transmission electron micrographs of human sperm cells (scale 500 nm). Normal ultrastructural pattern of a human spermatozoon, showing the nucleus (nu), acrosome (♦), equatorial segment (es), neck (ne), plasma membrane (pm), and outer and inner acrosomal membranes (OAM and IAM, respectively). The cytoskeletal structures at the principal and middle pieces are shown (axoneme, mitochondria, outer dense fibers, and fibrous sheet). The tail’s terminal piece is not shown (given that the cut section did not reach it). **(B)** Ultrastructural changes of the human sperm head. Electron micrographs shown in I–III illustrate different stages of the acrosome exocytosis. I. Sperm cell with an intact acrosome. II. Changes occurring upon receiving different stimuli, such as zona pellucida (ZP) or progesterone (Pg). A swollen acrosome with a waving outer acrosomal membrane (*) shows where membrane apposition occurs. III. Last stage (reacted). A sperm cell that has completed the acrosome reaction. Notice that the equatorial segment remains unaltered during the acrosome exocytosis. At the end of the reaction, the continuity between the plasma membrane and the outer acrosomal membrane is established, and the inner acrosomal membrane becomes part of the limiting membrane of the cell. A noticeable hybrid vesicle is still attached to the head.

The spermatozoon represents one of the most divergent cell types within the animal kingdom. In mammals, it is the smallest cell in the body and the only cell that performs its function in a different organism than where it was produced. After being formed in the seminiferous tubules of the testicle (spermatogenesis), sperm cells are conducted through the epididymal duct where they mature; once matured, the sperm cells are stored in the epididymal cauda until ejaculation, ultimately navigating through the female reproductive tract to reach the site of fertilization. The spermatozoon’s journey exposes it to numerous environmental, physical, and chemical barriers, along with selective pressures, which have driven its evolution toward deep morphological and functional specialization (Inaba, 2011). Therefore, the spermatozoon is a terminally differentiated cell characterized by its highly polarized structure. It contains a head and a tail or flagellum. The head exhibits distinct regions, namely, the acrosomal and postacrosomal regions, divided by the equatorial segment, with the nucleus and acrosome enclosed. The acrosome is a large granule that covers approximately half of the nucleus in the human sperm (Florman et al., 2008). The flagellum of mature sperm cells is in charge of cell movement, and it represents approximately 90% of the total length and contains the axoneme. This central structure runs throughout its entire length, starting at the neck region and ending at the terminal piece. It comprises a 9 + 2 arrangement of microtubules, sheath proteins, and mitochondria, ordered spirally only in the midpiece. In the following section, the principal piece, the mitochondrial sheath is absent and is replaced by a fibrous sheath. The terminal piece comprises a naked axoneme, and the microtubules end at different levels. The head and the tail are connected at the neck, enveloped by the plasma membrane with minimal cytoplasm inside. Sperm cells lack the capacity for protein or nucleic acid synthesis, and they have the sole purpose of locating, merging with, and transmitting genetic material to the oocyte for fertilization (Yanagimachi, 1994) (Figure 1).

The acrosome is an atypical secretory vesicle that goes through regulated exocytosis when stimulated by physiological inducers like progesterone or zona pellucida glycoproteins. The acrosomal membrane is partitioned into distinct segments, each serving unique roles. The segment positioned over the nucleus is dubbed the inner acrosomal membrane (IAM), while the region beneath the plasma membrane is termed the outer acrosomal membrane (OAM). It contains soluble acid hydrolases and other enzymes immersed in a dense protein matrix that makes it electron-opaque (Buffone et al, 2008) (Figure 1).

Millions of sperm ascend the uterus upon ejaculation in the female genital tract. However, only a few pass through the oviduct to reach the ampulla, where fertilization proceeds. This journey involves capacitation, a process comprising physiological, biophysical, and molecular changes, enabling the sperm to fertilize oocytes. Capacitated sperm acquire hyperactivated motility and the ability to release the acrosome content in the presence of physiological stimuli (Cohen et al., 2004; Gadella et al., 2008; Puga Molina et al., 2017; Stival et al., 2016; Visconti, 2009). The exocytosis of the sperm acrosome, known as acrosome reaction (AR), is modulated by Ca^2+^ and is necessary for oocyte fertilization (Belmonte et al., 2016; Buffone et al., 2014; Kirkman-Brown et al., 2002; Lishko et al., 2011; Michaut et al., 2000; Suhaiman et al., 2021; Suhaiman et al., 2010; Tomes, 2007; Trevino et al., 2006). The AR initiates a deep membrane rearrangement within the sperm head, and the acrosome undergoes striking morphological changes. In quiescent sperm, the acrosome typically exhibits dense electron content, positioned closely and parallel to the plasma membrane. The interaction between the OAM and PM occurs only under sperm activation upon stimulation by calcium ionophore or progesterone. Therefore, approximately 30%–40% of sperm display morphologically altered acrosomes (Sosa et al., 2015; Sosa et al., 2016; Zanetti and Mayorga, 2009). The acrosomes undergo swelling and waving on the OAM surface, which makes contact with the PM at multiple points, forming thousands of fusion pores and deep invaginations. A subset of cells demonstrates vesiculated or lost acrosomes, which is indicative of a reacted state. The AR is strongly regulated and irreversible. After the acrosome releases its contents, the cell undergoes a complete modification of its membrane components, exposing the IAM to the extracellular environment becoming the limiting membrane of the sperm while preserving the equatorial segment (Figure 1).

The AR involves the exocytosis of a huge granule with a dense matrix; therefore, whereas secretion in neurons and neuroendocrine cells is a quick process, the acrosome reaction requires several minutes to proceed after challenging sperm with different stimuli. During AR, Ca^2+^ increases with fast kinetics but the swelling of the acrosome preceding exocytosis is a slow process (t1/2 = 13 min). When swelling finishes, the fusion pore opening is fast. Acrosome swelling is the slowest step, and it defines the kinetics of acrosome secretion (Sosa et al., 2015; Sosa et al., 2016).

## 2 Unique lipids: the remarkable composition of human sperm membranes

The lipid composition of the sperm plasma membrane has been determined for different mammal species [for a review, see Gautier and Aurich (2022); Shan et al. (2021); Yanagimachi (1994). There are some differences between them, but in general, it is known that 70% are phospholipids, 25% are neutral lipids, and 5% are glycolipids (on molar base) of the total sperm plasma membrane lipids. Glycerophospholipids (GPLs) are the most representative molecules in the phospholipid fraction, with phosphatidylcholine and phosphatidylethanolamine being predominant. Notably, a significant proportion of these phospholipids comprises plasmalogens, which contain one fatty acid esterified with glycerol and an alkyl or alkenyl moiety forming an ether bond constituting two types of ether phospholipids, namely, plasmanyl (alkyl moiety at sn-1) and plasmenyl (alkenyl moiety with vinyl ether linkage at sn-1) (Tapia et al., 2012). Particularly, sperm membrane lipids contain a high proportion of polyunsaturated fatty acids such as arachidonic (20:4), docosapentaenoic (22:5), and docosahexaenoic acid (22:6) (Henkel, 2011). Another characteristic that distinguishes sperm membranes from somatic cell membranes is the presence of very long polyunsaturated fatty acids (VLC-PUFAs) within sphingolipids (Furland et al., 2007a; Furland et al., 2007b; Oresti et al., 2011). Remarkably, these polyenoic acyl moieties were of considerable length, ranging from 28 to 34 carbon atoms with 4–6 double bonds. The amount of double bonds makes sperm especially vulnerable to lipid peroxidation by reactive oxygen species (ROS) (Aitken et al., 2007). Regarding neutral lipids, sperm membranes mainly contain diacylglycerol (DAG) and cholesterol. Common glycolipid synthesis involves a glycosidic headgroup binding to ceramide (Sandhoff and Kolter, 2003). Still, in sperm, there is one notable exception: seminolipid, also known as the sulfogalactosylglycerolipid (SGG). This unique glycolipid, featuring a sulfo-galactosyl glycerol structure, is exclusively found in sperm membranes. It plays a crucial role in various stages of sperm development and function (Di Nisio et al., 2023; Gadella et al., 1995; Tanphaichitr et al., 2018). The main lipids building sperm membranes are summarized in Table 1 (Henkel, 2011; Sanocka and Kurpisz, 2004; Zalata et al., 2010; Zalata et al., 1998a; Zalata et al., 1998b).

**TABLE 1:** Main lipids of human sperm membranes (Henkel, 2011; Sanocka and Kurpisz, 2004; Zalata et al., 2010; Zalata et al., 1998a).

Component		nmol/10^8^ sperm	% of total fatty acids	%
Phospholipids				
Choline diacylglycerophospholipid		37.0	—	—
Ethanolamine diacylglycerophospholipid		31.5	—	—
Ethanolamine plasmalogen		20.0	—	—
Sphingomyelin		20.0	—	—
Choline plasmalogen		12.5	—	—
Fatty acid composition of phospholipids				
Saturated fatty acids (SFAs)			49.95	Of SFA
	Palmitic acid (C16)	105.5	29.73	59.52
	Stearic acid (C18)	35.9	11.35	22.72
Unsaturated fatty acids (UFAs)			50.05	Of UFA
	Docosahexaenoic acid (C22:6; (3)	108.0	21.54	43.04
	Oleic acid (C18:1; 09)	32.6	9.17	18.32
	Linoleic acid (C18:2; (6)	23.2	3.91	7.81
	Arachidonic acid (C20:4; (6)	20.1	2.39	4.77
	Icosatrienoic acid (C20:3; (06)	14.9	2.71	5.41
Sterols (neutral lipids)				
Cholesterol		133.0	—	—
Desmosterol		78.5	—	—
Glycolipids		6.4	—	—

Due to the absence of most cellular organelles and the DNA transcription machinery, as far as we know, sperm cells cannot synthesize lipids *de novo*. However, they possess an active and highly regulated system that keeps a dynamic refurbishing of sperm membranes adapting them for different functions throughout their lifespan. Membrane lipid remodeling occurs during epididymal sperm maturation (Dacheux and Dacheux, 2014; Flesch and Gadella, 2000; Sosnicki et al., 2023), capacitation (Asano et al., 2013b; Visconti, 2009), and the AR (Cheng et al., 2023; Lopez et al., 2012; Pelletan et al., 2015; Vaquer et al., 2020).

## 3 Cholesterol dynamics during acrosome exocytosis

Cholesterol is an abundant neutral lipid in sperm cells (Table 1) that plays a crucial role in modulating membrane properties during acrosome exocytosis. It is primarily synthesized by Sertoli cells in the testes and transferred by different mechanisms to spermatogenic cells (Shi et al., 2018). Cholesterol is distributed asymmetrically between the outer and inner leaflets of the plasma membrane. Typically, there is a slight enrichment of cholesterol in the outer leaflet compared to the inner leaflet (Ingolfsson et al., 2014). This asymmetry is crucial for maintaining the membrane’s structural integrity and functionality. Cholesterol can flip-flop between the inner and outer leaflets of the plasma membrane without the assistance of a transporter. However, this process is relatively slow compared to its lateral diffusion within the same leaflet. Transporter proteins, like ATP-binding cassette transporters, can facilitate and speed up this process, ensuring efficient cholesterol distribution across the membrane (Ogasawara and Ueda, 2022).

This sterol acts by influencing membrane fluidity, modulating the function of ion channels and key fusion proteins, and organizing lipid microdomains.

Due to deep lipid changes in the sperm plasma membrane accomplished during capacitation, the membranes become fusogenic and reactive to progesterone and zona pellucida glycoproteins (Harrison and Gadella, 2005; Spungin et al., 1992). Capacitation is a multifaceted and complex process within the female reproductive tract (Matamoros-Volante and Trevino, 2020; Orta et al., 2018; Stival et al., 2016; Visconti, 2009). Cholesterol loss is a hallmark of sperm capacitation, and a variety of functions have been assigned to this event that actively influences the function of the membrane during the AR. Cholesterol depletion starts shortly after sperm separation from seminal plasma. During the sperm transit in the female genital tract, cholesterol efflux occurs due to the presence of cholesterol acceptors such as high-density lipoproteins and albumin in the oviductal and follicular fluids (Bernecic et al., 2021; Bernecic et al., 2020; Ehrenwald et al., 1990; Leahy and Gadella, 2015). Sperm cells leap from an albumin concentration of 1 mg/mL (5 μM) in the semen (Elzanaty et al., 2007) to 34 mg/mL (500 μM) in the uterine fluid (Casslen and Nilsson, 1984). The human tubal fluid composition is usually mimicked in an *in vitro*-defined media to capacitate sperm artificially, and serum albumin is used as a cholesterol acceptor. During this process, the cholesterol content can decrease by up to 40% (Belmonte et al., 2005; Flesch et al., 2001; Flesch and Gadella, 2000). A direct consequence of cholesterol removal is that the plasma membrane fluidity of sperm cells increases and lateral redistribution of the sterol occurs toward the apical edge of the sperm head. These shifts in sperm lipid dynamics take place before the initiation of the AR (Companyo et al., 2007; Iborra et al., 2000; Leahy and Gadella, 2015).

**FIGURE 2:**
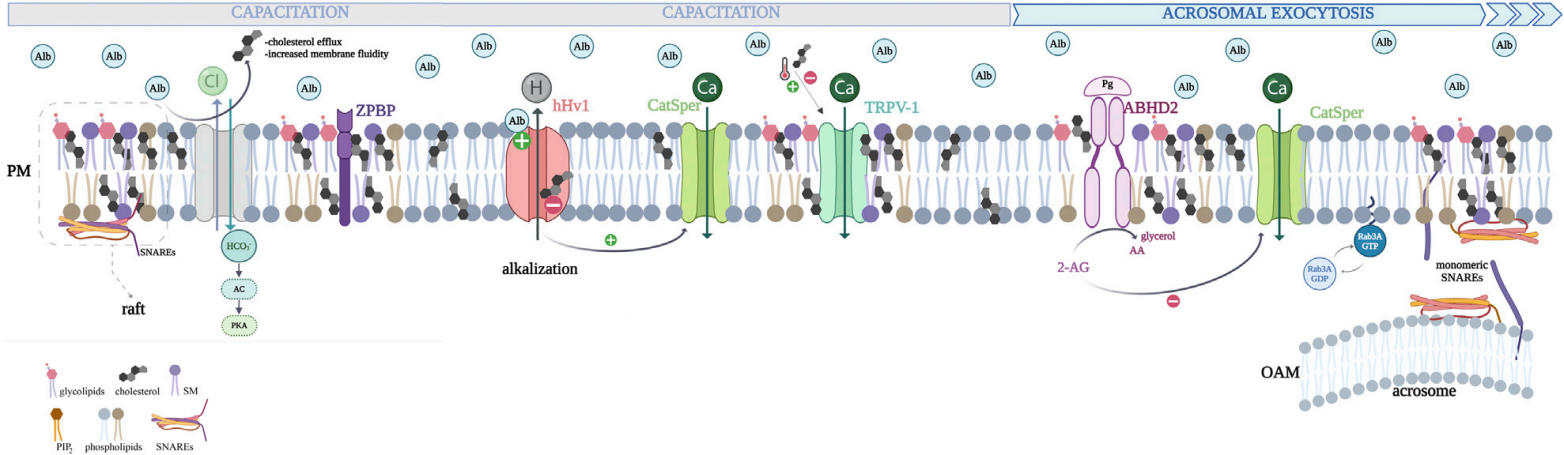
Schematic representation of the human sperm plasma membrane and changes induced by cholesterol efflux during capacitation and acrosome exocytosis. Major changes occur during capacitation, including increases in intracellular pH and stimulation of cAMP-dependent protein kinase (PKA). Cholesterol depletion is mediated by albumin in the female genital tract fluids (Alb, light blue circles). Albumin also binds to the human sperm proton channel, hHv1, activating it by interacting with its voltage sensor domains (VSDs). Its opening decreases the H^+^ concentration inside the human sperm, provoking alkalization required for the capacitation and consequently the AR. Cholesterol blocks the hHv1 channel function by stabilizing the voltage-sensing S4 at resting conformations. Thus, cholesterol efflux releases the channel from its inhibitory effect, favoring cytoplasmic alkalization. Cholesterol-depleted membranes become prone to facilitate sperm acrosome exocytosis via different complementary pathways. Cholesterol inhibits TRPV1 (transient receptor potential channel-vanilloid receptor 1) by interacting with an amino acid consensus sequence, which is known as the cholesterol recognition amino acid consensus (CRAC); on the other hand, it has been shown that mild temperatures activate this channel (thermotaxis). Cholesterol efflux allows the proper motility of sperm. Progesterone binds to and activates ABHD2 (progesterone receptor: α/β hydrolase domain-containing protein 2), a lipid hydrolase that cleaves the 2-AG (endocannabinoid 2- arachidonoylglycerol), which is a natural inhibitor of sperm CatSper (cation channel of the sperm). ABHD2 activation hydrolyzes 2-AG releasing glycerol and arachidonic acid (AA), opening CatSper and increasing [Ca^2+^]i. Recently, a computational-based approach suggested that cholesterol interacts directly with ABHD2 impairing progesterone-mediated sperm function through possible hydrolase inhibition. Cholesterol efflux allows the proper function of the receptor. Sphingolipids and cholesterol cluster in dynamic nanoscale domains embedded inside the plasma membrane phospholipid bilayer (lipid rafts). ZP-binding proteins are aggregated in the lipid-ordered fraction, as well as TRPV1 and ABHD2. Members of the SNARE family are concentrated in cholesterol-dependent domains: SNAP25 and syntaxin clusters. Certain molecules, such as isoprenylated proteins like the small GTPases, Rabs, tend to avoid lipid rafts and are generally excluded from these regions. The sterol content regulates its attachment to the membrane. When cholesterol levels decrease, active Rab3A can incorporate its geranylgeranyl groups into sperm membranes, recruiting effectors and initiating the tethering of the OAM to the plasma membrane. In summary, cholesterol efflux enhances fertilization competence by promoting the conditions necessary for the effective assembly of fusion machinery, leading to acrosome exocytosis.

Albumin has long been utilized as a cholesterol acceptor in sperm culture and *in vitro* fertilization (IVF) media (Balaban et al., 2014); however, Zhao et al. (2021) demonstrated that in addition to having this function albumin binds to the human sperm proton channel, hHv1, activating it by interacting with the voltage sensor domains (VSDs). Its opening decreases the H^+^ concentration inside human sperm, inducing alkalization required for the capacitation and consequently the AR (Zhao et al., 2021) (Figure 2).

As stated before, cholesterol also plays a role in regulating channels and protein activity. Next, we will examine this function in sperm. Dr. Travis’s group demonstrated that the cholesterol efflux and focal enrichment of the ganglioside GM1 induce murine sperm calcium influx through the CaV2.3 channel (Cohen et al., 2014), triggering calcium transients required for AR [reviewed in (Cohen et al., 2016)]. The cholesterol decrease also results in proteolytic activation by a furin-class protease of phospholipase B (PLB) that hydrolyzes both fatty acids from phospholipids. The products may alter membrane curvature, favoring membrane fusion during physiologically induced AR in mouse sperm (Asano et al., 2013a; Asano et al., 2013b).

Recently, Han et al. (2022) demonstrated that cholesterol hinders the hHv1 channel function by stabilizing the voltage-sensing S4 at resting conformations. Therefore, cholesterol efflux releases the channel from its inhibitory effect, inducing human sperm cytoplasmic alkalization (Han et al., 2022). Furthermore, these authors showed that arachidonic acid reverses cholesterol inhibition (Han et al., 2023).

Moreover, the transient receptor potential channel-vanilloid receptor 1 (TRPV1) regulates, partly, sperm-oriented motility to reach the oocyte (Bahat et al., 2003). De Toni et al. (2021) demonstrated that in human sperm, cholesterol inhibits TRPV1 by interacting with an amino acid consensus sequence, known as the cholesterol recognition amino acid consensus (CRAC), present in the channel S5 helix domain (Picazo-Juarez et al., 2011).

Recently, the same group described the mechanism by which membrane cholesterol regulates progesterone-mediated sperm activation. Membrane cholesterol levels are linearly and inversely correlated with the progesterone-induced effects in human sperm cells, such as increases in [Ca^2+^]i, chemotaxis, motility changes, and AR. Progesterone binds to and activates ABHD2 (progesterone receptor: α/β hydrolase domain-containing protein 2), a lipid hydrolase that cleaves the endocannabinoid 2-arachidonoylglycerol (2-AG), releasing glycerol and arachidonic acid (AA) and increasing [Ca^2+^]i (Miller et al., 2016). The 2-AG is a natural inhibitor of sperm CatSper (cation channel of sperm). CatSper mediates hyperactivated motility during capacitation (Lishko et al., 2011), and the calcium current it produces is required for the AR (Vaquer et al., 2023; Vaquer et al., 2020). A computational-based approach suggested that cholesterol interacts directly with ABHD2 impairing progesterone-mediated sperm function through possible hydrolase inhibition. Molecular dynamic analysis indicates that cholesterol binding affects the conformational freedom of key amino acids involved in the binding of the enzyme substrates (De Toni et al., 2023) (Figure 2). Cholesterol efflux allows the proper function of the progesterone receptor.

We will discuss another function of cholesterol: its role in forming microdomains, which may influence the localization of exocytosis-related proteins by determining the most suitable membrane regions for their activity. Sphingolipids and cholesterol clusters in dynamic nanoscale domains embedded within the phospholipid bilayer (lipid rafts) (Brown and Rose, 1992; de Almeida et al., 2003). Lipid rafts demonstrate less fluidity than the neighboring membrane (Calder & Yaqoob, 2007; Lingwood et al, 2009). Since 2001, biochemical evidence and high-resolution imaging support a direct role for cholesterol in exocytosis, given that members of the SNAREs (soluble N-ethylmaleimide-sensitive factor attachment protein receptors), like syntaxin 1 and synaptosome-associated protein 25 (SNAP25), are concentrated in cholesterol-dependent domains, known as SNAP25 and syntaxin clusters (Chamberlain et al., 2001; Lang, 2007; Lang et al., 2001; Weisgerber et al., 2024). The presence of SNAREs in lipid rafts concentrates these proteins at precise plasma membrane zones. R-SNAREs like synaptobrevin (VAMP) and Q-SNAREs like syntaxin, as well as the Ca^2+^ sensor protein synaptotagmin, are enriched in sperm-derived lipid rafts from different mammal species, including humans (Gadella et al., 2008; Gamboa and Ramalho-Santos, 2005; Lv et al., 2008; Nixon and Aitken, 2009; Nixon et al., 2011). The complementary SNARE proteins, syntaxin, and VAMP have been localized to the apical region of the mouse sperm head (Brahmaraju et al., 2004; Tsai et al., 2007; Zitranski et al., 2010) and sperm cells of other species. In murine and guinea sperm cells, Syntaxin 2 can be found in both raft and non-raft fractions (Asano et al., 2009; Selvaraj et al., 2006; Travis et al., 2001). Importantly, molecules involved in sperm-zona pellucida binding have been recovered from detergent-resistant membrane preparations (DRM) obtained from capacitated sperm (Zitranski et al., 2010). The sperm SNARE proteins, including VAMP from the outer acrosomal membrane and syntaxin from the apical plasma membrane of the sperm head, exhibited similar lateral redistribution characteristics during capacitation. The same redistribution pattern was also observed in the ZP-binding protein complex and raft marker proteins (Boerke et al., 2008). Interestingly, sperm SNAREs and ZP-binding proteins are aggregated in the same lipid-ordered fraction of the sperm head, and this is relevant for ZP-binding and ZP-induced AR (Zitranski et al., 2010) (Figure 2).

Due to the physical properties of cholesterol microdomains, proteins can distribute themselves among various membrane regions. Certain molecules, such as isoprenylated proteins like the small GTPases, Rabs, tend to avoid lipid rafts and are generally excluded from these regions (Pyenta et al., 2001). The effect of cholesterol efflux has been extensively studied during capacitation. Our group proposed that cholesterol might directly influence the AR. To investigate this, we induced acute cholesterol depletion in capacitated human sperm just before stimulating them with progesterone. This cholesterol removal resulted in the increased occurrence of the AR. Cholesterol seems to affect an early step of the exocytic cascade rather than the mixing of lipid bilayers. One specific target of cholesterol’s effect is Rab3A. The sterol content does not influence the activation–deactivation cycle of Rab3A but regulates its membrane attachment. Our results suggest that initially, sperm SNAREs concentrate in lipid rafts, while Rab3A remains in the cytosol. However, when cholesterol levels decrease, active Rab3A can incorporate its geranylgeranyl groups into sperm membranes, recruiting effectors and initiating the tethering of OAM to the plasma membrane. In summary, cholesterol efflux enhances fertilization competence, by promoting the conditions necessary for the effective assembly of fusion machinery, leading to acrosome exocytosis (Belmonte et al., 2005) (Figure 2). Thus, cholesterol is a critical regulator of sperm AR.

## 4 Bioactive synergy: the power of sphingolipids in exocytosis

Sphingolipids represent ∼10–20% of total membrane lipids (Bartke and Hannun, 2009). Sphingolipid’s *de novo* synthesis pathway starts exclusively with the activity of a serine palmitoyl transferase (SPT) from serine and palmitate which forms the first sphingolipid, ceramide. This last sphingolipid can further be synthesized via the cleavage of sphingomyelin (SM) as a result of the sphingomyelinase (SMase) activity (SM pathway). The metabolism ends with S1P lyase, which degrades sphingosine-1-phosphate (S1P) into non-sphingolipid molecules. *De novo* synthesis of sphingolipids occurs in the endoplasmic reticulum [for a review, see Gault et al. (2010); Hannun and Obeid (2008)]. The intermediary steps form a complex network connecting various sphingolipids. In this network, ceramide acts as a central core, given that it is the only one synthesized *de novo* and can originate all the sphingolipids of the pathway (Petrache et al., 2013; Silva et al., 2012; Sridevi et al., 2009). The sphingolipids ceramide, S1P, and ceramide 1-phosphate (C1P) are critical signaling molecules regulating numerous physiological functions such as exocytosis [reviewed in Gomez-Munoz et al. (2016)].

A defining trait of the plasma membrane (PM) in mammalian cells is the asymmetric distribution of lipids across the bilayer (Doktorova et al., 2020). Sphingomyelin (SM) is the main sphingolipid in the outer leaflet and is present in membrane nanodomains alongside cholesterol. SM has two aliphatic chains and a zwitterionic headgroup. Hence, it only occasionally flip-flops across bilayers. However, Abe et al. showed that SM might move from the outer leaflet to the inner leaflet of the PM by a mechanism involving a peripheral myelin protein 2 (PMP2), which is a phosphatidylinositol 4,5-bisphosphate (PIP_2_)-binding protein. They showed that PMP2 provokes the tubulation of membranes in a PIP_2_-dependent manner, coupled with changes in sphingomyelin transbilayer localization (Abe et al., 2021). In secretory cells, SM is considered mostly a structural lipid that can release metabolites considering highly bioactive molecules (Gafurova et al., 2024; Tsentsevitsky et al., 2023).

**FIGURE 3:**
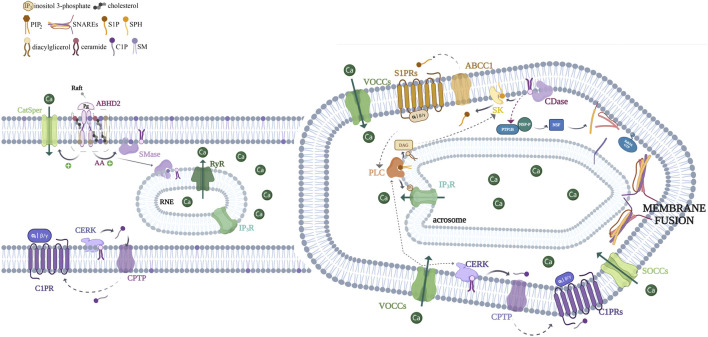
Schematic representation depicting the role of sphingolipids in acrosome exocytosis. We present here a working model hypothesized for the sphingolipid pathway involved in the acrosome exocytosis. Progesterone interacts with its receptor (α/β hydrolase domain-containing protein 2, ABHD2) to trigger lipid hydrolysis, leading to the generation of arachidonic acid (AA), an activator of neutral sphingomyelinase (nSMase). In turn, this enzyme hydrolyzes sphingomyelin to produce ceramide. However, it remains possible that nSMase triggers the pathway, and due to that, it might also be activated by other stimuli (AA from other sources, reactive oxygen species, and anionic phospholipids) (Airola and Hannun, 2013). As already known, the activation of ABHD2 leads to the stimulation of the sperm cation channel (CatSper). This occurs because ABHD2’s lipid hydrolase activity, associated with the progesterone receptor, cleaves the endocannabinoid 2-arachidonoylglycerol (2-AG), thereby releasing CatSper from its inhibition. This allows the calcium current through CatSper, which is required for hyperactivated motility during capacitation and later for the AR. Endogenous ceramide exerts various effects, for example, it activates CatSper and ryanodine sensitive-calcium channels (RyR) located in the redundant nuclear envelope (RNE), contributing to calcium mobilization toward the sperm head. On the other hand, the first calcium wave activates ceramide kinase (CERK), leading to the C1P synthesis. Given that C1P-induced AR requires CatSper activity, we suppose that the sphingolipid can set off CatSper via an unknown, alternative mechanism or by using the same machinery utilized by Pg. The last possibility makes sense due to the CERK activity requirement for Pg-triggered exocytosis. The CERK phosphorylates ceramide to C1P, which can be transported through the plasma membrane to the extracellular environment by a C1P transfer protein (CPTP). Extracellular C1P interacts with a Gi-coupled receptor (C1PR) activating VOC channels and a heterotrimeric Gi-protein. In the same manner, S1P is generated by the phosphorylation of sphingosine by the action of sphingosine kinase 1 (SK1). The S1P is probably transported to the extracellular space by the ATP-binding cassette family member of transporters named ABCC1, where it interacts with a Gi-coupled receptor (S1PR). Both GPCR pathways lead to calcium increase and the heterotrimeric Gi-protein switch on a PLC, which hydrolyzes phosphatidylinositol 4,5-bisphosphate (PIP_2_) producing DAG and IP_3_. The last one binds to IP_3_-sensitive calcium channels, which is present on the outer acrosomal and redundant nuclear envelope (RNE) membranes, inducing calcium efflux from the reservoirs. The voiding of the stores triggers the opening of SOCCs at the plasma membrane, allowing a sustained calcium increase. On the other hand, ceramide activates a signal transduction cascade involving the activity of protein tyrosine phosphatase 1B (PTP1B), which dephosphorylates NSF (N-ethylmaleimide-sensitive factor) and elicits the SNARE (SNAP receptor) complex disassembly during human sperm exocytosis. Both a local increase in calcium coming from IP_3_-sensitive channels and SNAREs converge to accomplish the final steps of membrane fusion. The SK/S1P pathway seems to follow a pathway independent of an acute ceramide increase.

Focusing on the sperm of different species, during the steady state, *Xenopus*, bull, ram, rat, and boar sperm exhibit notably high levels of SM (approximately 12–20 mol%) (Stith, 2015). However, in rodents and boar sperm, SMase activity increases just before fertilization hydrolyzing SM, mainly containing VLC-PUFA, to produce ceramides during the AR, with a consistent SM decrease (Ahumada-Gutierrez et al., 2019; Nikolopoulou et al., 1986a; Nikolopoulou et al., 1986b; Penalva et al., 2018; Petcoff et al., 2008; Zanetti et al., 2010a; Zanetti et al., 2010b). Cross (2000) reported that exogenous SMase accelerates capacitation by promoting sterol loss and ceramide production in human sperm. As a result, sperm become responsive to progesterone stimuli, releasing the acrosome content. The sperm SM likely serves a primarily structural role and acts as a source of ceramide that exerts its specific biological activity (Figure 3). Murase et al. (2004) reported that ceramide increased the AR induced by calcium ionophore in boar sperm cells. Given that the effect of ceramides on secretory cell exocytosis is controversial (Abousalham et al., 1997; Ji et al., 2011; Mansfield et al., 2004; Tang et al., 2007; Won et al., 2018) and the molecular mechanism has not been fully explained, our group focused on the role of ceramide during the AR. Ceramide contains two aliphatic chains and a neutral headgroup, enabling it to flip-flop within membranes. However, for ceramide to move between different membranes, it requires the presence of vesicles or specific transfer proteins. The ceramide transfer protein (CERT) plays a crucial role in transporting ceramide from its synthesis site to the required location (Hannun and Obeid, 2008). Vaquer et al. (2020) provided a comprehensive exploration of the molecular mechanisms initiated by ceramide during acrosome exocytosis. The study identifies and localizes key sphingolipid metabolism enzymes—such as nSMase, ceramide synthase, and neutral ceramidase—in human sperm, highlighting both basal and regulated ceramidase activity during the AR (Vaquer et al., 2020). The neutral ceramidase degrades ceramide into sphingosine and free fatty acids. It is localized to the plasma membrane and the endoplasmic reticulum membrane in somatic cells, where it functions optimally at a neutral pH. Endogenous and exogenous ceramide leads to a rapid and early rise in intracellular calcium levels involving at least three channels: CatSper channels, ryanodine receptors (RyR), and store-operated calcium channels (SOCCs). This initial calcium wave activates a PLC, which hydrolyzes PIP_2_ into inositol 3-phosphate (IP_3_) and DAG. IP_3_ then promotes the release of intracellular calcium stores by activating IP_3_-sensitive calcium channels (IP3R), leading to prolonged SOCC opening and sustained calcium augment, which drives the acrosome exocytosis.

Although ceramide synthase (CerS, Lass) is found in the acrosomal region of human sperm (Vaquer et al., 2020), we do not believe that ceramides can be synthesized “*de novo*” in a terminal cell lacking the endoplasmic reticulum, where this process occurs in somatic cells. Instead, evidence suggests that the increase in ceramides during the AR is due to the activity of nSMases present in the inner leaflet of the plasma membrane (Vaquer et al., 2020).

Ceramide increases in human sperm activate protein tyrosine phosphatase 1B (PTP1B), resulting in the dephosphorylation of the N-ethylmaleimide-sensitive factor (NSF) and subsequent disassembly of the SNARE complex. The study also reveals that both progesterone and ceramide activate PTP1B and necessitate VAMP2 for the AR, so both pathways converge in the final steps of membrane fusion. This study provides compelling evidence for the critical pathways regulated by ceramide during sperm acrosome secretion, clarifying the debated role of this lipid in exocytosis signaling pathways (Vaquer et al., 2020).

Due to the sperm sphingolipid metabolism appearing very active and the ability of ceramide to be converted into all sphingolipids in the pathway, the acute ceramide increase might be exerted by itself or by its generated products. Among the molecules with biological activities related to exocytosis, S1P and C1P are the most attractive candidates. They are bioactive lipids, meaning that, slight concentration changes can have significant functional consequences. S1P and C1P, far from structural lipids, are present in minimal concentrations, undergo rapid turnover, and achieve signaling function. S1P has a zwitterionic headgroup, including a charged phosphate, and is likely to move freely among membranes but is unlikely to flip-flop spontaneously. S1P acts as an intracellular second messenger or extracellularly by binding to its receptors. S1P interacts with five G-protein coupled receptors (S1PR1-5) that belong to the lysophospholipid receptor family (Spiegel and Milstien, 2003).

Our laboratory has made significant strides in understanding the S1P signaling mechanisms underlying human sperm acrosome exocytosis. S1P induces the AR in human sperm by activating a Gi-coupled receptor (Suhaiman et al., 2010), leading to [Ca^2+^]i increase, driven by extracellular ion influx through VOCCs and SOCCs, as well as release from intracellular stores via IP3Rs. The pathway’s efficacy relies on the activity of phospholipase C (PLC), protein kinase C (PKC), and the small GTPase Rab3A, which inserts into sperm membranes recruiting effectors to initiate membrane fusion. Furthermore, we demonstrated the presence and activity of sphingosine kinase 1 (SK1) in human sperm cells. DAG and its analog, the phorbol ester PMA, induce SK1 translocation/activation from the cytosol to the membrane and S1P synthesis. Importantly, exocytosis induced by PMA is dependent on SK1 activity. Our findings reveal that PMA stimulates S1P synthesis, which can be transported extracellularly, indicating the existence of an S1P transporter. The extracellular S1P then binds to specific Gi-coupled receptors, triggering the signaling cascade which results in AR. Thus, S1P can induce the AR through an autocrine/paracrine effect (Belmonte and Suhaiman, 2012; Suhaiman et al., 2010). Our work highlights the crucial role of the SK1/S1P/S1PR signaling pathway in regulating acrosome secretion, as depicted in Figure 3.

Since ceramide stimulates the AR (Vaquer et al., 2020) and is a near-direct precursor of S1P, which is synthesized by human sperm cells inducing acrosome secretion (Suhaiman et al., 2010), we hypothesized that an acute rise in ceramide could exert its effect on exocytosis through the synthesis of S1P. However, an increase in ceramide does not provoke S1P synthesis in sperm cells and induces exocytosis in an S1P-independent manner (Vaquer et al., 2023). Instead, the rise in ceramide leads to the production of C1P (Vaquer et al., 2023). C1P carries ionic charges at neutral pH and has two hydrophobic chains. It likely remains in its synthesis compartment and is unlikely to flip spontaneously across bilayers. The C1P synthesis in human sperm implies the presence of CERK. Our group demonstrated the existence of an active CERK in human sperm, further showing that its activity is regulated by calcium. C1P induces exocytosis in capacitated human sperm cells, causing a rise in [Ca^2+^]i that involves calcium influx from the extracellular medium and calcium efflux from internal reservoirs. Ceramide induces the AR, primarily due to C1P production. Notably, progesterone required the activity of CERK to evoke a [Ca^2+^]i increase and acrosome exocytosis. Our research is pioneering in describing the molecular mechanism triggered by the bioactive sphingolipid C1P in the physiological progesterone pathway that triggers membrane fusion during sperm AR. In conclusion, the physiological activity of progesterone in human sperm depends on ceramide (Vaquer et al., 2020) and C1P (Vaquer et al., 2023) but is S1P-independent (Suhaiman et al., 2010) (Figure 3).

In light of the findings discussed, we propose a mechanism in which C1P interacts with a C1P receptor in the human sperm plasma membrane, inducing signaling. C1P is produced intracellularly as CERK catalyzes ceramide phosphorylation in a calcium-dependent manner within the sperm. The bioactive sphingolipid can be transported to the extracellular medium by a C1P transfer protein (CPTP) (Presa et al., 2020). It may then bind to a putative Gi protein-coupled receptor. Despite the partial characterization of a potential C1P receptor, the receptor has not yet been cloned or isolated (Arana et al., 2013; Ouro et al., 2013). We suggest that the progesterone present in the female tubal fluid induces a calcium influx through CatSper by activating α/β hydrolase domain-containing protein 2 (ABHD2) (Miller et al., 2016). This increase in calcium stimulates the CERK in human sperm cells to produce C1P, triggering the mechanism described (Figure 3).

We hypothesize that the physiological stimulus eliciting S1P signaling is the oocyte zona pellucida and that it should be produced from ceramide. An acute rise in ceramide induces C1P synthesis, whereas progesterone requires both ceramide and C1P—but may bypass the S1P pathway—to trigger acrosome exocytosis.

In conclusion, sphingolipids play a pivotal role in membrane remodeling during membrane fusion, and maintaining a precise balance of these lipids in human sperm is essential for successful fertilization.

## 5 Diacylglycerol (DAG)

Phosphoinositide-specific PLC catalyzes the cleavage of PIP_2_ into DAG and IP_3_. Non-specific PLC generates DAG by hydrolyzing phosphatidylethanolamine (PE) or phosphatidylcholine (PC) (Sim et al., 2020). DAG induces the fusion of liposomes and is required to merge organelle membranes (Fratti et al., 2004; Jun et al., 2004). It induces vesicle fusion through various mechanisms, such as those mediated by TRP channel activation (Albert, 2011). DAG and PMA significantly enhance neurotransmitter release and promote exocytosis in PC12 cells (Xue et al., 2009). They bind to and activate C1-domain-containing proteins, such as PKCs and Munc13, the latter being a prime factor for SNARE-dependent exocytosis (Houy et al., 2022; Rhee et al., 2002; Trexler and Taraska, 2017). Recently, the reconstitution of complete fusion protein machinery demonstrated a new function for DAG in vesicle priming and the cargo release rate (Sundaram et al., 2023).

**FIGURE 4:**
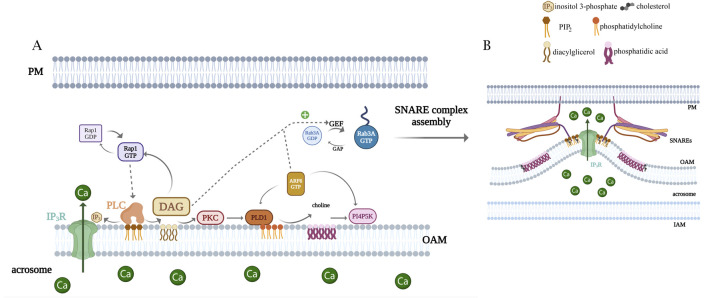
Model highlighting the importance of lipids as signaling molecules for the AR. **(A)** Rap1 GDP/GTP exchange is promoted by DAG and/or Epac1 and triggers PLC epsilon activation, generating the loop shown in the cartoon. DAG also activates PLD1 via PKC. Active PLD1 hydrolyzes phosphatidylcholine, generating choline and PA. The latter could be activating phosphatidylinositol 4-phosphate 5-kinase (PI4P5K), which catalyzes PIP_2_ and closes a positive feedback loop. PIP_2_ hydrolysis generates IP_3_, which elicits the efflux of calcium from the acrosome. Simultaneously, DAG connects with the proteinaceous membrane fusion machinery by activating Rab3A, which leads to the assembly of trans SNARE complexes. Exocytic stimuli also promote the GDP/GTP exchange in ARF6 that stimulates PLD1, PI4P5K, and PLC activities, driving PIP_2_ turnover and contributing to the lipid cascade. **(B)** There is a remarkable bending of the outer acrosomal membrane associated with acrosomal swelling. We postulate a significant role for cone (PA)- and inverted cone (PIP_2_)-shaped lipids during the AR. Lipid modifications in the cytosolic hemileaflet of the OAM during the exocytotic stimulus may induce changes in the membrane curvature that may foster the growing of deep invaginations of the OAM and the recruitment of SNARE proteins.

DAG and phorbol esters induce the AR in sperm from different species. The endogenous sperm synthesis of DAG is regulated by physiological agonists (Murase and Roldan, 1996; O’Toole et al., 1996a; O’Toole et al., 1996b; O'Toole et al., 1996c; Vazquez and Roldan, 1997). DAG activates TRP channels in the sperm plasma membrane, inducing the Ca^2+^ influx from the extracellular medium (Jungnickel et al., 2001; Jungnickel et al., 2007). This lipid can also impact other factors involved in exocytosis. Given the diverse actions of DAG, our group investigated whether this lipid plays further roles after the opening of TRP calcium channels localized in the sperm plasma membrane. To achieve this, we performed selective plasma membrane permeabilization with streptolysin O (SLO), abrogating ion gradients to dissect DAG additional intracellular actions (Lopez et al., 2012). We developed this protocol specifically for human sperm (Bello et al., 2012; Bustos et al., 2012; Diaz et al., 1996; Lopez et al., 2007; Michaut et al., 2001; Roggero et al., 2005). Our research demonstrated that DAG/PMA induces exocytosis without the extracellular calcium requirement, instead necessitating calcium release from internal stores via IP_3_-dependent channels and the presence of functional SNAP-25. DAG activates a signaling cascade by stimulating PKC, which activates PLD1 to synthesize phosphatidic acid (PA). This PA drives the production of PIP_2_ and phosphatidylinositol-(3,4,5)-trisphosphate (PIP_3_) (Figure 4). The exocytosis triggered by DAG and calcium converges within the same signaling pathway. Therefore, DAG sustains elevated IP_3_ levels through a positive feedback loop that results in the continuous generation of PIP_2_. Additionally, DAG stimulates a GTPase exchange factor (GEF) for Rab3A, activating it and enabling the tethering of the plasma membrane to the OAM, facilitating protein interactions that drive the SNARE complex assembly and membrane fusion.

## 6 Tiny but mighty: the diverse functions of small phospholipids

### 6.1 Phosphatidylinositol-4,5-bisphosphate (PIP_2_)

In eukaryotic cells, PIP_2_ is produced in the inner leaflet of the plasma membrane by the highly regulated activity of kinases and phosphatases that modify phosphoinositides (Olivenca et al., 2018). It constitutes less than 2 mol% of the total phospholipid content and is also present in vesicle membranes. Unlike structural lipids, PIP_2_ exists in minimal quantities and undergoes rapid turnover, which makes it particularly suited for signaling roles (Delage et al., 2013).

PIP_2_ plays multiple roles during exocytosis by clustering into platforms that delineate membrane domains, attracting proteins from the cytosol and membrane and concentrating them in a signaling spot. PIP_2_ signaling is transient and localized. It functions as the first and second messenger in signal transduction and molecular recognition processes, and it shapes membranes poised to fuse. Membrane fusion necessitates the transformation of flat lipid bilayers into highly curved structures, making membrane bending a crucial force. In general, PIP_2_ assembles into membrane microdomains affecting the membrane curvature and tension (Bradley et al., 2020; Slochower et al., 2013; Wang et al., 2014). The effect is due to its inverted cone shape, where the hydrophobic portion occupies the less surface area than the head [for a review consult (Bura et al., 2023; Martin, 2012; Martin, 2015)]. PIP_2_ clusters induce powerful electrostatic interactions with positively charged molecules (McLaughlin and Murray, 2005; Williams et al., 2009) and recruit proteins containing specific PH domains involved in exocytosis (Martin, 2012; Martin, 2015). Calcium, through its positive charge, bridges reversibly syntaxin 1/PIP_2_ complexes into mesoscale domains (Milovanovic et al., 2016); therefore, physiological [Ca^2+^] drives transient lipid plasma membrane reorganization for secretion. The synaptic vesicle calcium sensor, synaptotagmin, binds to PIP_2_ regulating exocytosis (Park et al., 2015), e.g., phosphoinositide enhances 40-fold the affinity of synaptotagmin-1 for calcium (Honigmann et al., 2013; van den Bogaart et al., 2012). Ji et al. (2017) found that local PIP_2_ metabolism is essential for vesicle tethering and docking. PIP_2_ regulates different types of channels including voltage-gated K^+^ channels, inward-rectifier K^+^ channels, sensory transduction channels, and voltage-gated Ca^2+^ channels (Hille et al., 2015; Suh and Hille, 2005; Suh et al., 2012; Suh et al., 2010).

Physiological inducers of the AR elicit a transient and quick calcium entry from the extracellular space into the cytosol, which is crucial for phospholipase activation. Then, PI-PLCs bind PIP_2_ through their specific PH domain, hydrolyzing the substrate during an early step in the AR [for a review, see Roldan and Shi (2007)]. Our laboratory unveiled a new cascade occurring after the first calcium wave during the acrosome exocytosis (Figure 4), where PIP_2_ hydrolysis occurs again at a later step, but the phosphoinositide is re-synthesized entering a feedback cycle (Lopez et al., 2012).

Our research has provided compelling evidence showing that ADP-ribosylation ractor 6 (ARF6) is present in human sperm cells, undergoing GDP to GTP exchange in response to exocytic signals and subsequently governing lipid exchange (Pelletan et al., 2015). ARF6 alternates between a GTP, membrane-bound “on” state, and a GDP-bound, cytosolic “off” state. During the activation phase, a guanine nucleotide exchange factor (GEF) releases GDP bound in the nucleotide pocket, and ARF binds GTP. This is the rate-limiting step in ARF activation. During the inactivation phase, hydrolysis converts GTP to GDP, catalyzed by ARF’s intrinsic GTPase activity assisted by GTPase-activating proteins (GAPs). ARF6 regulates actin cytoskeleton reorganization and different membrane trafficking pathways. Suzuki et al. (2009) demonstrated the presence of EFA6, an ARF6-GEF, in spermatogenic cells of adult mouse testes. Proteomic analysis of the human sperm revealed that ARF6 is present in the male gamete and different GAPs and GEFs for these GTPases (Wang et al., 2013). Given that we demonstrated that exocytic stimuli in human sperm induce changes in their lipid profile (Belmonte et al., 2016; Lopez et al., 2012; Pelletan et al., 2015), and, in particular, that PIP_2_ and PIP_3_ levels increased, we hypothesize that both lipids contribute to ARF6-GEFs binding to membranes via their pleckstrin homology (PH) domain. Although we previously reported that calcium and DAG activate a GEF for ARF6 (Pelletan et al., 2015), we have not yet detected the presence of GEFs for ARF6 in human sperm cells.

Upon GTP binding, ARF6 activates PLC, which hydrolyzes PIP_2_-producing DAG and IP_3_-reliant intra-acrosomal calcium release. Additionally, ARF6, once activated, stimulates PLD1 and PIP kinase, resulting in increased levels of PA and PIP_2_, respectively. The small GTPase promotes phospholipid remodeling for the continuous synthesis and hydrolysis of PIP_2_. ARF6-GTP is the master modulator of the feedback loop and consequently the lipid turnover essential for acrosome exocytosis. Concomitantly, ARF6 activates Rab3A, driving the fusion protein machinery assembly, becoming the OAM and PM physically attached (Pelletan et al., 2015). Our study unveils the molecular connection involving ARF6, PLC, PLD, PIP kinase, and Rab3A, shedding light on the intricate interplay between lipids and proteins during AR (summarized in Figure 4).

### 6.2 Phosphatidic acid (PA)

Three alternative routes are known to synthesize the signaling lipid PA. First, the DAG kinase (DGK) catalyzes the phosphorylation of DAG to produce PA. Second, the acylation of lysophosphatidic acid (LPA) is driven by specific LPA-acyltransferases (LPAAT) producing PA (Jenkins and Frohman, 2005). Third, PLD catalyzes the hydrolysis of the distal phosphodiester link of PC generating PA and choline. PLDs are mainly responsible for PA synthesis during exocytosis. Mammals have six PLD isoforms, with PLD1 and PLD2 functioning as lipases involved in exocytosis. PLD2 is constitutively active and PLD1 activity is highly regulated by different stimuli (Bowling et al., 2021; Frohman, 2015; Humeau et al., 2001; Zeniou-Meyer et al., 2007).

PA contains a tiny headgroup, negatively charged with two fatty acids. Due to this chemical structure, it adopts a conical shape. A localized build-up of PA in a single membrane leaflet can alter membrane topology by inducing a negative curvature in *in vitro* experiments (Kooijman and Burger, 2009; Kooijman et al., 2005a; Kooijman et al., 2005b). One theory posits that PA plays a crucial role in the secretion process by stabilizing the intensely curved structure of the fusion pore during membrane fusion (Chernomordik and Kozlov, 2005; Chernomordik and Kozlov, 2008). Nevertheless, the exact timing and location of PA formation during exocytosis remain unknown. PA is a main signaling molecule and forms microdomains via intermolecular hydrogen bonds, recruiting proteins to their proper location to achieve their role (Tanguy et al., 2020; Tanguy et al., 2021; Tanguy et al., 2022). There are a lot of data involving PLD1/PA in the fusion of secretory vesicles during regulated exocytosis in neurons and neuroendocrine cells; however, the molecular mechanism is not clear enough [for a review, see Ammar et al. (2013); Chasserot-Golaz et al. (2010); Tanguy et al. (2021); Tanguy et al. (2022); Vitale, (2010)]. Most results point out its effect on the membrane curvature, and some results indicate that PA modulates the membrane penetration of dynamin (Barber et al., 2018; Raben and Barber, 2017). Lam et al. (2008) showed that syntaxin-1A interacted with PA and defined a polybasic region within this protein considered as the lipid-binding domain.

During bovine sperm capacitation, a PLD activity is necessary for actin polymerization (Cohen et al., 2004; Cohen et al., 2016). Bates et al. (2014) described that in *Xenopus laevis* sperm, PLD is activated to increase PA 2.7-fold by 1 min after insemination. The inhibition of PA synthesis abolished PLCγ and tyrosine kinase Src activation. Our laboratory showed that PLD1/PA is part of a signaling cascade that sustains PIP_2_/IP_3_ synthesis. PLD is present in human sperm cells and that DAG activates the enzyme, leading to PA increase through PKC activation. PA is indispensable for membrane fusion during the AR (Lopez et al., 2012). The activity of PLD1 is highly regulated by active ARF6 in acrosome exocytosis (Belmonte et al., 2016; Pelletan et al., 2015). Another protein that interacts directly with PA is Epac1 (Consonni et al., 2012). Epac1 is a cAMP-modulated guanine nucleotide exchange factor for the small G-protein Rap. Epac and Rap are present in the human sperm and connect the cAMP signal (Branham et al., 2009; Branham et al., 2006; Lucchesi et al., 2016) to lipid synthesis shown in the loop described (Figure 4).

Considering that the membranes cannot stretch or deform easily, how do the invaginations form? Where do the lipids that create these invaginations originate? We propose that PIP_2_ (inverted-cone) and PA (cone) are synthesized mainly in the OAM, clustering in microdomains, which induce membrane bending that originates invaginations (Figure 1). This synthesis causes the OAM to wave during acrosome swelling. This hypothesis is illustrated in Figure 4.

## 7 Discussion

The WHO defines infertility as a disease characterized by the inability to achieve pregnancy after 12 months of unprotected sexual intercourse (https://www.who.int/news-room/fact-sheets/detail/infertility). It is estimated that there are 186 million people worldwide with infertility, and with current trends, it is projected that there will be two million more cases per year (Szkodziak et al., 2016). Male reproductive failure accounts for half of couples’ infertility (Agarwal et al., 2015). The causes of male infertility include numerous factors: genetic, endocrine, male reproductive system pathologies, systemic diseases, and lifestyle factors (Durairajanayagam, 2018). Idiopathic infertility is diagnosed in men with a normal spermogram after excluding all contributing factors, and it accounts for up to 15% of male reproductive dysfunction (Tvrda et al., 2021).

This review underscores lipid balance and dynamic changes in sperm membranes must be tightly regulated to guarantee physiological processes. Increasing attention is focused on how paternal factors influence fertilization and embryo development (Vallet-Buisan et al., 2023). The lipid composition of the membrane holds sperm structure and function and could be related to altered semen analysis and fertilizing ability. Research on how sperm lipid composition affects male fertility remains insufficient. The results clustered here closely link with data demonstrating that infertile patients with abnormal sperm parameters presented augmented levels of membrane cholesterol (Chen et al., 2021; Di Nisio et al., 2023; Force et al., 2001; Garolla et al., 2018; Hou et al., 2023; Samavat et al., 2014; Sedes et al., 2018). As previously stated, sperm membrane SMs contain high levels of VLC-PUFA. Recently, a study on human sperm found a correlation between the health of the human male gamete and VLC-PUFA concentration, suggesting that sperm quantity and quality possibly depend on these fatty acid levels (Craig et al., 2019).

Some studies have allowed the identification of sperm lipid markers related to mammalian fertility (Shan et al., 2020; Shan et al., 2021). In a preliminary investigation, a lipid cluster has been recognized to differ significantly between fertile and infertile men and associated with the semen parameters. Indeed, SGG, cholesterol sulfate, and PUFAs represented the most important lipid markers to predict semen quality (Di Nisio et al., 2023). Validating these results and tuning up a routine analysis of lipid markers for the laboratory of assisted reproductive technology (ART) might be helpful in predicting the ability of sperm samples to render successful FIV (*in vitro* fertilization) and ICSI (intra cytoplasmic sperm injection) procedures. A deeper understanding of how paternal factors transported from sperm cells to embryos contribute could illuminate ways to enhance ART from an andrological standpoint.

Future research is required to determine whether there is a correlation between the sperm lipid profile and different reproductive outcomes, such as sperm health, fertilization rates in FIV and ICSI procedures, pregnancy, and live birth. A reliable parameter is urgently required because the current standard semen analysis has low accuracy in predicting the sperm-fertilizing capability and male fertility. The next challenge would be to analyze whether sperm membrane lipid homeostasis can be restored by supplementing the diet or culture media with the correct factors. If successful, such interventions could offer a revolutionary approach to the treatment of male infertility, providing hope to millions of couples worldwide.
